# Translation, extension, and evaluation of usability, usefulness, and safety of a fall prevention and management program for people living with spinal cord injury and multiple sclerosis who use wheelchairs or scooters full time

**DOI:** 10.3389/fresc.2024.1406938

**Published:** 2024-09-18

**Authors:** Laura A. Rice, Malaak Yehya, Jennifer Yi, Stephen Koziel, Elizabeth W. Peterson

**Affiliations:** ^1^ Department of Health and Kinesiology, University of Illinois Urbana-Champaign, Champaign, IL, United States; ^2^ Department of Occupational Therapy, University of Illinois Chicago, Chicago, IL, United States

**Keywords:** wheelchair, falls, self-management, online education, community participation

## Abstract

**Background:**

Falls are prevalent among people living with Multiple Sclerosis (PwMS) and Spinal Cord Injury (PwSCI) who use wheelchairs or scooters (WC/S) full time, however, there is a scarcity of evidence-based fall prevention and management programs.

**Objective:**

To describe the systematic translation of an in-person fall prevention and management program (Individualized Reduction Of FaLLs – iROLL) for PwMS to an online platform, extending its scope to include PwSCI, and to evaluate the preliminary useability, usefulness, and safety of the intervention.

**Methods:**

iROLL was systematically translated to an online platform (iROLL-O). PwMS and PwSCI who use a WC/S full time, experienced at least one fall within the past 36 months, and could transfer independently or with minimal to moderate assistance, enrolled in iROLL-O. Usability, usefulness, and safety were evaluated through 1:1 semi-structured interviews, gathering feedback on: perceived impact of the intervention on falls and functional mobility, program experiences, adverse events, and recommendations for improvement.

**Results:**

Five participants successfully completed the iROLL-O program. No safety concerns were raised by participants. Themes emerging from the semi-structured interviews included: (1) barriers and facilitators to program access, (2) motivation for participation, (3) program outcomes, and (4) program content and structure. Participants reported reduced concerns about falling, enhanced functional mobility skills, and highlighted the supportive nature of synchronous group meetings for learning.

**Conclusion:**

No adverse events occurred during the implementation of iROLL-O and participants found the program to be useable and useful. Further testing is needed to examine efficacy among a large and diverse population.

## Introduction

Approximately 50%–80% of persons living with Spinal Cord Injury (PwSCI) (1) and 25% with Multiple Sclerosis (PwMS) (2) use wheelchairs or scooters (WC/S) full-time (>75% of mobility is via a WC/S) to support mobility activities. Between 69%–75% of these populations report at least one fall over a 12-month period (3) and 65%–75% express concerns about falling and limitations due to this concern (3). Falls and concerns about falling can substantially impact an individual's community engagement and quality of life (4). Activity curtailment, often driven by concerns about falling, can hinder societal roles and compromise engagement in daily living activities (4), which can lead to diminished access to important health information. Furthermore, the cycle of disuse and deconditioning can potentially increase fall risk in these populations (5).

Despite the high frequency and significant concerns associated with falls among PwSCI and PwMS who use WC/S, evidence-based fall prevention and management programs are scarce. The individualized reduction of falls (iROLL) program was developed to serve PwMS who use WC/S full-time. iROLL is a group-based intervention delivered by physical (PTs) or occupational therapists (OTs) that utilizes the Health Belief Model and Social Cognitive Theory as its theoretical foundation. iROLL is designed to mitigate fall incidence and concerns about falling. Moreover, it aims to improve knowledge concerning fall risks and functional mobility skills. The overarching objectives include enhancing quality of life and promoting community participation.

iROLL was first implemented in-person (iROLL-IP). While findings indicate that iROLL-IP is an efficacious program (6), during pilot testing approximately 20% of participants either declined to participate or dropped out of the study due to challenges related to transportation to/from the intervention sessions, despite the intervention being held in accessible locations. Lessons learned during implementation of the iROLL-IP program (6, 7), as well as the need for online programing to support vulnerable populations, prompted efforts to translate the iROLL program to an online format, iROLL-Online (iROLL-O). Due to the abrupt end of in-person testing caused by the COVID-19 pandemic, the iROLL-IP program was quickly adapted to an online platform to complete an ongoing study (8). This adaptation lacked a systematic process.

In addition, iROLL-IP was originally designed for PwMS who use WC/S full time. Due to the high frequency of falls, significant consequences, and lack of peer-reviewed education for PwSCI, a pressing need exists to expand iROLL-O to include participants living with SCI.

The purpose of this paper is twofold. The primary purpose is to describe the systematic translation of iROLL to an online platform and extension of scope to include PwSCI who use WC/S full time. Additionally, it seeks to evaluate the usability, usefulness, and safety of iROLL-O through end-user feedback. We hypothesize that iROLL-O will be found to be useable and useful by PwMS and PwSCI who use WC/S full time and safe to implement.

## Methods

### Translation of iROLL to an online format and inclusion of PwSCI

The adaptation process was guided by the Intervention Mapping Protocol (IMP) framework (9). The IMP is a multiple step, systematic approach use to develop theory- and evidenced-based health promotion programs. The initial step of IMP involves the performance of a needs assessment and development of a logic model. Feedback from iROLL-IP and pilot iROLL-O participants and the PTs and OTs who administered the intervention, were gathered to examine the facilitators and barriers of the program. Additionally, critical program components that elicited change and reasons behind participants non-enrollment were explored. A comprehensive overview of the those findings is described and can be found in Van Denend et al. (7, 10). In addition, parallel feedback was obtained from PwSCI regarding their desired fall prevention and management needs (11). Utilization of a group format, as well as incorporation of an interdisciplinary team were identified as pivotal aspects of iROLL implementation. Suggested changes included items such as providing examples of downgraded exercises, adding home safety assessments, and modifications.

During the second step of the adaptation process, matrices of change objectives were prepared. The change objectives captured participant feedback on program and training changes while outlining how changes in one's desired behavior would be accomplished.

The third step of the IMP involves selecting a theoretical model and program design. The Health Belief Model (HBM) (12) and Social Cognitive Theory (SCT) (13) served as the theoretical foundation for iROLL-IP and were deemed appropriate for iROLL-O. Key components of the HBM informing iROLL-O include perceived susceptibility to falls, perceived benefits of fall management, and fostering participant self-efficacy. The SCT constructs utilized include self-regulation and self-efficacy in one's motivation and learning. Practical considerations included program time requirements and opportunities for practice in a safe and supportive manner. After the initial design of the online program was developed, feedback was obtained from our program advocate, a physician living with MS who participated in iROLL-IP, through semi-structured interviews and integrated into the design of the program.

The fourth step involved developing program materials and the iROLL-O website. Modifications were made to the iROLL-IP written instruction materials based on participant feedback and the matrix of change. In addition, a team of PTs and OTs with SCI experience carefully reviewed and expanded program content by integrating additional education materials that cater to the needs of PwSCI. Next, the iROLL-IP materials were translated to an online format following best practices associated with online learning (14). Website development was informed by the Web Accessibility Initiative (WAI) (www.w3.org) guidelines. The research team worked closely with professional videographers and web designers to produce the iROLL-O content though an iterative process until a consensus was reached among team members. The program advocate provided formal feedback through a semi-structured interview after reviewing the program in its entirety. All materials associated with iROLL-O can be accessed here: https://iroll.kch.illinois.edu/.

### iROLL -O program structure

iROLL-O is an online, six-week, fall prevention and management education program that consists of two components: (1) asynchronous independent learning opportunities and (2) synchronous online discussions with peers led by clinicians. Each week, participants review the asynchronous materials and engage in a synchronous online discussion.

The asynchronous independent learning opportunities comprise pre-recorded, video-based education modules. Intervention topics areas are informed by circumstances associated with falls (15, 16) including: wheelchair/scooter skills, transfer skills, core and upper extremity exercises, environmental hazard management, post-fall recovery, assistive technology upkeep and utilization, and home modifications. These modules include extensive use of videos and images, including peer modeling of functional mobility skills. Like iROLL-IP, multiple versions of the education content are provided to accommodate diverse participant needs. For example, education is provided on both independent and assisted transfer techniques. Participants are guided through the process of establishing short and long-term goals and encouraged to engage in weekly journaling. Action planning strategies are utilized to facilitate the integration of new education materials into daily routines.

Synchronous online discussions, conducted weekly via the platform Zoom (San Jose, CA), are led by a PT or OT. Following a manualized session guide, the PT or OT leading the session will address questions from participants and pose reflection prompts to encourage group dialogue. Participants also can engage in social interactions with peers and receive peer feedback on challenges faced.

### Assessment of useability, usefulness, and safety

A qualitative research study was conducted using semi-structured interviews to examine the useability, usefulness, and safety of iROLL-O among PwMS and PwSCI who use WC/S full-time. All study related procedures were approved by the Institutional Review Board at the University of Illinois Urbana-Champaign.

## Recruitment

Participants were recruited from across the United States between May and August 2021 through online disability support groups, social media, and the University of Illinois Urbana-Champaign Disability Resources and Educational Services research registry. Our recruitment goal was set at 6 participants, *a priori*. This sample size was selected to allow for an in-depth, focused analysis of the participants to inform future, large-scale studies.

All interested individuals were invited to participate in the study if they met the following inclusion criteria: (1) a diagnosis of MS or SCI; (2) 18-years-old or older; (3) full-time use of a WC/S (>75% of mobility is via a wheelchair or scooter); (4) self-reported ability to transfer independently or with minimal/moderate assistance, and (5) at least 1 self-reported fall in the past 36 months. Individuals were excluded if (1) they were unable to remain in an upright position for at least an hour; (2) were not agreeable or did not have access to a care partner that could assist when practicing the skills taught in the intervention; (3) did not have access to the internet or an internet-capable device; (4) had an MS exacerbation in the past 30 days (if applicable), or (5) received a score of 10 or above on the Short Blessed Test (17).

## Study design

**Figure 1:**
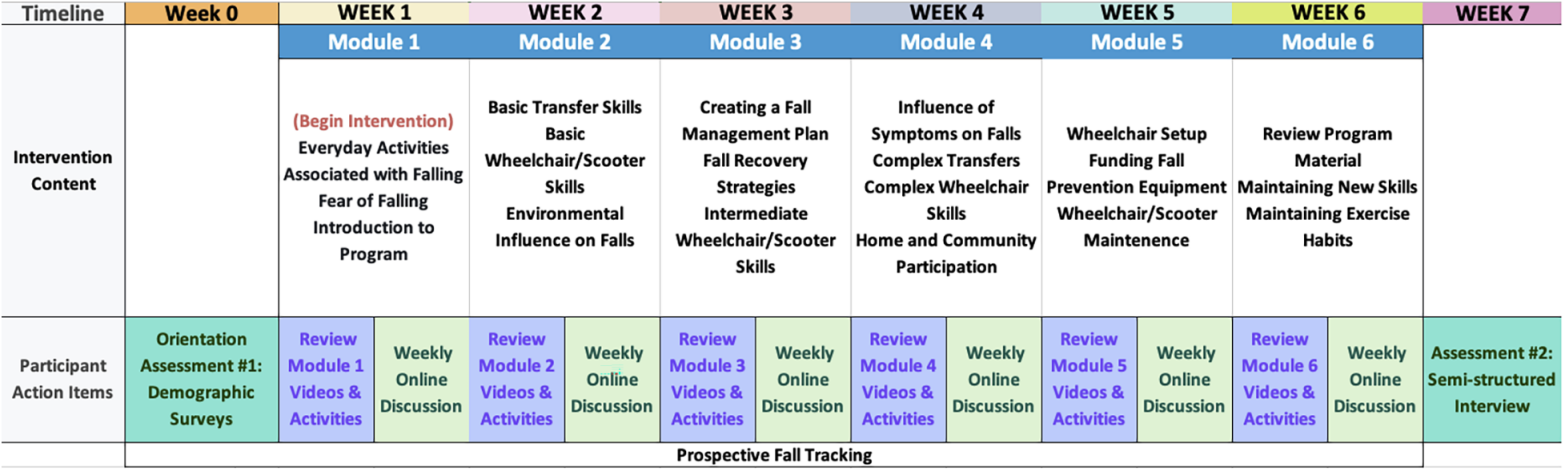
iROLL-O study design.

An overview of the study design is presented in Figure 1.

### Screening and study orientation

All research activities were performed online and data was collected through a secure, online survey platform, Research Electronic Data Capture (REDCap). All participants provided informed consent and underwent eligibility verification by a trained research assistant. Subsequently, a trained research assistant conducted an online technology screening to examine the feasibility of accessing iROLL-O from the participant's home, including testing their ability to engage in a video conference and stream program-related videos. If participants lacked sufficient technology resources, the research assistant attempted to troubleshoot technology barriers. If attempts were unsuccessful, the participant was withdrawn from the study. Participants with adequate technology received a 30-min orientation to the iROLL-O program. Moreover, care partners were instructed on how to assist the study participant during the education program.

### Assessment #1

Next, participants completed an online assessment (assessment 1) via a REDCap to gather information on the participant's history of MS or SCI and basic demographic information.

### iROLL-O intervention

After completion of assessment 1, participants engaged in the iROLL-O intervention, as described in the section “Structure of the iROLL-O Intervention”. The synchronous sessions were delivered by a trained PT (author L.R.) with over 15 years of experience providing care to PwSCI and PwMS who use a WC/S to groups of 2–3 participants over the course of six weeks.

### Assessment #2

After completion of the intervention (approximately 7 weeks post assessment 1), participants engaged a 20–30-min, Zoom-based, semi-structured interview with a trained member of the research team to collect feedback on the intervention materials, perceived influence of the intervention on falls and functional mobility, positive and negative program experiences, adverse events, and recommendations for change. Interviews were recorded with permission of the participants. Interview questions are provided in Table 1.

**Table 1:** Interview guide.

The purpose of the interview is to ask your thoughts and feedback about the online iROLL program. This interview will help us to examine the program. Your feedback is critical in this process and will help support the ongoing development of the program. Any feedback and thoughts you provide are valued and appreciated.
1. Please tell me why you chose to participate in this program?
2. Please describe your overall experiences with the program. Can you think of a specific story or incident that characterizes the overall experience?
3. Was this program helpful to you in your everyday life, why or why not?
4. Do you feel the frequency of your falls increased, decreased or stayed the same? Why do you feel this way?
5. Do you feel that your transfer and/or wheelchair/scooter skills improved or became more refined as a result of the education program? Why do you feel this way?
6. What are your feelings about fear of falling and fall management after completing this program?
7. Were there any challenges or barriers to your participation in this program?
8. How did you feel about the structure of the program that included independently viewing pre-recording materials and then meeting up with a therapist to discuss the materials and having the opportunity to ask questions about the materials?
9. Have you made any changes in your life to prevent or manage falls after completing this program?
10. Since completing this program, have there been any changes to your participation in daily activities that are meaningful or important to you?
11. Is there anything else that you would like to tell me about this program that I have not asked you about?

## Statistical analyses

Quantitative analysis was performed with SAS software (SAS version 9.4, SAS Institute Inc., Cary, NC, 2018) to examine central tendency of demographic data.

Recordings of the semi-structured interviews were transcribed verbatim. Each transcript was analyzed by two members of the research team, independently. Research team members were a graduate and undergraduate student with prior qualitative research experience in the Department of Health and Kinesiology. A thematic analysis framework (18) was used to guide the structure of the analysis to develop key themes. In addition to maintaining a reflexive approach, providing thick descriptions and utilizing an audit trail, two research team members used a shared code book to conduct independent open coding. The code book was iteratively refined to address coding discrepancies and newly emerging codes until a consensus was reached. All final-coded transcriptions were verified by a third member of the research team, a senior researcher with a background in Rehabilitation Science, who was not involved in the initial code development. Ongoing data analysis was performed, and demonstrative quotations were identified upon creation of the codebook.

## Results

Participants who completed the intervention were an average of 51 ± 16 years old, 60% male (*n* = 3), 60% lived with SCI (*n* = 3), and 60% use a manual chair as their main form of mobility (*n* = 3). See Table 2 for additional details.

**Table 2:** Baseline participant characteristics.

Participant	Age	Gender	Health condition	Mobility device	Falls in past 6 months	Primary mobility device use (hours/week)	Years since disability onset
1	61	Female	SCI	Manual wheelchair	8	100	50
2	24	Male	SCI	Manual wheelchair	4	168	7
3	50	Male	MS (Secondary progressive)	Power wheelchair	4	100	24
4	57	Female	SCI	Manual wheelchair	2	80	36
5	62	Male	MS (Relapsing- remitting)	Power wheelchair	1	60	17

Six participants enrolled, meeting our recruitment goal. One participant withdrew after completing the first assessment citing time constraints. Five participants began the intervention and successfully completed it. Four participants completed semi-structured interviews; one participant was lost to follow-up. No adverse events were recorded during the implementation of the intervention. All participants reported having a care partner to support them. One fall was reported during the study period, but it was not associated with any study related activities and no injuries were reported.

Table 3 shows the common themes from interviews of participants who completed the intervention. Four main themes, with associated sub-themes and codes, emerged: (1) barriers and facilitators to program access, (2) motivation for participation, (3) program outcomes, and (4) program content and structure.

**Table 3:** Post-intervention participant interview themes, sub-themes, & codes.

Theme	Sub-theme	Codes
Barrier and facilitators	Facilitators	
motivation for participation	Fall and injury prevention	
	Strength and skill improvement	
	Fall recovery	
Program outcomes	Overall outcomes	Heightened awareness
		Task modification
	Perception of fall frequency and fear of falling	Decreased
		No change
	Perception of functional mobility skills	Improved
		No change
	Perception of participation in meaningful activities	No change
Program content and structure	Comprehensive program	
	Foundational knowledge	
	Synchronous sessions	Shared experience/peer interaction
		Active participation
	Areas for improvement	In-person sessions/hands on instruction
		More advanced techniques
		More participant accountability
		Mobile app

### Barriers and facilitators to program access

All participants were able to engage with the study and had sufficient internet access to participate. No significant barriers associated with participating in the online meetings or steaming asynchronous videos was noted.

“It was nothing that took a whole lot of time to do. I don't think anything really inhibited me from doing it.” – Participant 3, Power Wheelchair User, Male

The website and structure of the program manual were noted as facilitators to program engagement.

“the course materials online was really easy to access. I didn't have any problems with it. I like how you guys put [the exercise program] at the back of the program manual… So, it’s easier for us to just copy the exercises, [and] you don't have to flip pages.” – Participant 2, Manual Wheelchair User, Male

### Motivation for participation

In response to questions exploring their motivation for participating in iROLL, participants reported a desire for fall and injury prevention, as well as hopes to improve strength, functional mobility skills, and fall recovery techniques:

…any information to help myself or other people to avoid falling out of their chair and injuring themselves seemed like a worthy goal.” – Participant 5, Power Wheelchair User, Male

…wheelchair skills, and to learn how to transfer back to my wheelchair. - Participant 2, Manual Wheelchair User, Male

### Perceptions of program outcomes

With respect to participants’ perceptions of the program, participants discussed overall outcomes of the program and reported gaining heighted awareness of the potential for a fall.

“I really try to visualize what I'm going to do before I do it. And I think that helps, when.the fall gets in your head.” – Participant 3, Power Wheelchair User, Male

“I definitely focus more on cracks in the sidewalk and looking for potential hazards.” - Participant 5, Power Wheelchair User, Male

Participants also discussed how they modify tasks.

When I'm reaching for a high object, [I] take a minute before I reach outside of my… cylinder of balance,… there is potential for a fall here, and we make sure I'm braced against something. It’s what keeps coming back.” –Participant 5, Power Wheelchair User, Male

Participants also provided more specific details about the impact of iROLL-O on fall frequency, fear of falling, and function mobility. Some participants felt that their fall frequency and fear of falling decreased because of the program.

“So it decreased [fear of falling], because I learned the techniques on how to get back to my wheelchair.” – Participant 2, Manual Wheelchair User, Male

Others did not perceive a change in fear of falling, but did report an increased awareness of the need for safety:

“I don't think my fear of falling has changed. I think it’s consistent with the way it was before. I definitely don't want to [fall], but it has made me realize.…to ask for help, or to wait.” – Participant 3, Power Wheelchair User, Male

Participants also noted improvements in their functional mobility skills and how mobility skills related to fall risk:

"I felt like it really got me to think about my whole process of transferring and to think it through before I did it, even if I was in a hurry.” – Participant 3, Power Wheelchair User, Male

Finally, participants were asked about the program's influence on facilitating participation in meaningful activities. Participants highlighted the impact of the COVID-19 pandemic to explain why changes in participation did not occur:

“No, but COVID did have a definite impact…unfortunately, no one could do anything.” – Participant 1, Manual Wheelchair User, Female

### Program content and structure

Participants were asked to provide feedback on useability and usefulness with emphasis on program content and structure. Participants felt the program was comprehensive and covered a lot of information within a condensed timeframe.

“I thought the program was spot on, it touched on a lot, covered a lot in the six weeks, and I thought it was well put together, well thought out, well planned- the timing [and] sessions getting together.” – Participant 1, Manual Wheelchair User, Female

Participants also appreciated that different options provided for the exercise program and transfer skills.

“I think that the way they presented it as giving you different levels of difficulty, you could do it with a weight or you could do it without. I think that that it gave you some alternatives…if you're tired that day… then you can still do it.” – Participant 3, Power Wheelchair User, Male

Participants noted the benefit of learning foundational insights into the cause of falls, providing them with a valuable perspective.

“I think that what [the intervention has] done is it’s made me think about [my falls] a little bit more. When I did fall [I questioned] why it happened and analyzed what caused the fall” – Participant 1, Manual Wheelchair User, Female

The synchronous sessions were highlighted by the participants as a positive aspect of the program. They emphasized that the shared experiences and peer interactions were great opportunities for learning.

“I loved how we did a group setting, because each of us had very different types of disabilities. So it was good to learn from the others that were in the group on how they go about doing things.” – Participant 1, Manual Wheelchair User, Female

Participants explained that the synchronous sessions contributed to increasing active participation.

“I liked that we had a meeting with a face to go with a program. And it wasn't just all book, and, you know, even just a voice, just a call, I like that it was Zoom, so that I could see and talk to other people that way.” – Participant 3, Power Wheelchair User, Male

Regarding areas for improvements, participants reported a desire for learning more advanced techniques:

“I want to learn how to transfer from the tub to my wheelchair.” – Participant 2, Manual Wheelchair User, Male

The desire for more participant accountability was also noted:

“If I had some ongoing group or some sort of incentive or something to keep me focused, perhaps would have been more incorporated into my routine.” – Participant 5, Power Wheelchair User, Male

Finally, participants reported that having a mobile application would help to improve ease of access to program materials:

“I wish that there's like an app where people can access the course materials….” – Participant 2, Manual Wheelchair User, Male

## Discussion

This paper describes the systematic translation of iROLL from an in-person program to an online platform, along with the extension of the scope of the program to include PwSCI. In addition, the usability, usefulness, and safety of iROLL-O was evaluated.

The systematic translation using the IMP assured the program retained the effective elements of the in-person program. Online health education has been found to improve health literacy, a critical factor in understanding health conditions and maintaining a healthy lifestyle in the long term (19), and improving the emotional state and satisfaction of end-users (20). Flexibility in access to education can improve outcomes (14) and is of particular importance for PwSCI and PwMS as they are often affected by pain, fatigue, or have limited activity tolerance. In addition, online health education can help improve the emotional state and satisfaction of end-users (19). Mitzner, et al. indicates that adults aging with long-term mobility impairments were accepting of the idea of using tele-technology to participate in remote physical exercise classes and engage in social interaction (20).

In this study, no barriers associated with internet access, engagement with study materials, participation in online discussions, or navigating the website were identified.

Participant safety was a priority, and no safety concerns arose. All participants had a care partner available to assist during practice of physical skills. The iROLL program aims to refine existing skills rather than teach entirely new skills. Similarly, Worobey, et al. (17) did not encounter any adverse events when investigating efficacy of an online transfer training program among 71 people using power and manual wheelchairs full-time. During the synchronous discussions, participants seeking to learn new skills requiring significant hands-on support were encouraged to seek in-person therapy services. Participants received guidance on self-advocacy for communication with health care providers about specific skill goals. Further testing with a larger, more diverse population is needed to comprehensively explore potential technological barriers and assess the availability of care partners to provide support.

Participants reported heightened awareness regarding falls and their management, including recognition of fall-prone situations and environmental factors that can contribute to falls. The ability to analyze a fall experience and understand why it had happened was also noted. Participants also discussed increased confidence in performing functional activities without fear of falling and recovering from falls. Post-fall management is critically important as pervious research has found that extended time on the ground after a fall can lead to death or injury and increases the risk of hospital and long-term care admissions (21). Similar results were observed among PwMS in a pilot iteration of iROLL-O, where reports of fear of falling was significantly reduced and an increase in perceived ability to manage risk of falls and knowledge of fall prevention and management were found (8).

While perceived improvements in functional mobility skills were reported, participants reported a desire to learn more difficult skills. While the online platform has extended reach, it limits hands-on instruction and practice. Future iterations may consider optional in-person sessions to refine functional mobility skills or collaboration with local therapists to provide individualized, hands-on instruction.

Participants did not report a perception of improvement in meaningful activities post-intervention. After engaging in iROLL-IP, improved community participation was noted (6). Although we are unable to prove a causal link, it is likely that the COVID-19 lockdown affected these measures, and this hypothesis is supported by participants responses to the semi-structured interview questions. Barriers to in-person activities due to the COVID-19 pandemic exposed a clear need for an at-home, online program that provided fall prevention and management education. While activity restrictions associated with the COVID-19 pandemic have lifted, effective online programing is still needed due to limited access to knowledgeable professionals, lack of transportation resources, and seasonal periods of increased health risk due to viruses and other infectious diseases.

Overall, participants were very positive in their feedback about iROLL-O and emphasized the value of group sessions, in particular, as a way to learn from the OT or PT and fellow group members. Feedback from iROLL-IP (6) and iROLL-O (8) participants has consistently highlighted that participants appreciate iROLL's group format and value skilled facilitators (7, 10). In the future, participants suggested an app-based system for easier access to study materials and to foster communication.

## Limitations

This study was a small (*n* = 5) study. Evaluating the intervention via a randomized trial involving a larger participant pool is necessary to comprehensively assess implementation within a community setting and the intervention's effectiveness. Future trials should prioritize underrepresented populations to explore how factors like age, gender, socioeconomic status, and cultural background impact program experiences and outcomes.

This study was conducted in 2021 during the COVID-19 pandemic. Given the need to limit community involvement for health and safety reasons, the lack of findings associated with quality of life and community participation should be considered within this context. Further examination of the impact of iROLL-O on quality of life and community participation is warranted.

Given that these findings were self-reported by participants, it is likely that social desirability bias potentially affected the validity of the results. It is also important to consider the impact that response bias has on both the initial screening and reported improvements.

## Conclusions

iROLL-O, an online fall prevention and management intervention, was successfully translated from an in-person program to an online platform, expanded in scope to support PwSCI, and found to be useable, useful, and safe for PwSCI and PwMS who use WC/S full time. Participants reported increased confidence in fall prevention and management, heightened environmental awareness, and improved fall risk identification. This study highlights the value and potential of online education in fall prevention, particularly for people with limited transportation resources and access to knowledgeable healthcare professionals. Future studies with larger, more diverse samples are needed to examine the efficacy of iROLL-O.

## Data Availability

The raw data supporting the conclusions of this article will be made available by the authors, without undue reservation.

## References

[1] National Spinal Cord Injury Statistical Center. Annual Statistical Report for the Spinal Cord Injury Model Systems- Complete Public Version. Birmingham, Alabama: University of Alabama at Bimingham (2021).

[2] Wallin MT Culpepper WJ Campbell JD Nelson LM Langer-Gould A Marrie RA The prevalence of MS in the United States: a population-based estimate using health claims data. Neurology. (2019) 92(10):e1029–40. 10.1212/WNL.0000000000007035 30770430 PMC6442006

[3] Sung J Trace Y Peterson EW Sosnoff JJ Rice LA. Falls among full-time wheelchair users with spinal cord injury and multiple sclerosis: a comparison of characteristics of fallers and circumstances of falls. Disabil Rehabil. (2019) 41(4):389–95. 10.1080/09638288.2017.1393111 29069956

[4] Sung J Shen S Peterson EW Sosnoff JJ Backus D Rice LA. Fear of falling, community participation, and quality of life among community-dwelling people who use wheelchairs full time. Arch Phys Med Rehabil. (2021) 102(6):1140–6. 10.1016/j.apmr.2020.11.013 33347892

[5] Mazumder R Lambert WE Nguyen T Bourdette DN Cameron MH. Fear of falling is associated with recurrent falls in people with multiple sclerosis: a longitudinal cohort study. Int J MS Care. (2015) 17(4):164–70. 10.7224/1537-2073.2014-042 26300702 PMC4542711

[6] Rice LA Yarnot R Sung J Sosnoff JJ Backus D Abou L Pilot study of a fall prevention and management intervention program for people with multiple sclerosis who use a wheelchair or scooter full-time. Arch Rehabil Res Clin Transl. (2022) 4(4):100225. 10.1016/j.arrct.2022.100225 36545518 PMC9761259

[7] Van Denend T Peterson EW Sung J Rice LA. Process evaluation findings of a fall prevention and management program for wheelchair and scooter users with multiple sclerosis. PEC Innov. (2022) 1:100081. 10.1016/j.pecinn.2022.100081 37213774 PMC10194268

[8] McArthur AR Peterson EW Sosnoff J Backus D Yarnot R Abou L Online delivery of the individualized reduction of falls intervention for persons with multiple sclerosis who use a wheelchair or scooter full-time: a pilot study. Int J MS Care. (2023) 25(2):82–90. 10.7224/1537-2073.2022-044 36923574 PMC10010107

[9] Bartholomew LK Parcel GS Kok G. Intervention mapping: a process for developing theory- and evidence-based health education programs. Health Educ Behav. (1998) 25(5):545–63. 10.1177/109019819802500502 9768376

[10] Van Denend T Peterson EW Sung J Rice LA Process evaluation findings of a fall prevention and management program for wheelchair and scooter users with multiple sclerosis. PEC Innov. (2022) 1:100081. 10.1016/j.pecinn.2022.100081 37213774 PMC10194268

[11] Rice LA Sung JH Keane K Peterson E Sosnoff JJ. A brief fall prevention intervention for manual wheelchair users with spinal cord injuries: a pilot study. J Spinal Cord Med. (2020) 43(5):607–15. 10.1080/10790268.2019.1643070 31343950 PMC7534352

[12] Rosenstock IM Strecher VJ Becker MH. Social learning theory and the health belief model. Health Educ Q. (1988) 15(2):175–83. 10.1177/109019818801500203 3378902

[13] Bandura A. Self-efficacy: toward a unifying theory of behavioral change. Psychol Rev. (1977) 84(2):191–215. 10.1037/0033-295X.84.2.191 847061

[14] Müller C Mildenberger T Steingruber D. Learning effectiveness of a flexible learning study programme in a blended learning design: why are some courses more effective than others? Int J Educ Technol High Educ. (2023) 20(1). 10.1186/s41239-022-00379-x 36811132 PMC9934945

[15] Rice LA Peters J Sung J Bartlo WD Sosnoff JJ. Perceptions of fall circumstances, recovery methods, and community participation in manual wheelchair users. Am J Phys Med Rehabil. (2019) 98(8):649–56. 10.1097/PHM.0000000000001161 31318744

[16] Rice LA Sung J Peters J Bartlo WD Sosnoff JJ. Perceptions of fall circumstances, injuries and recovery techniques among power wheelchair users: a qualitative study. Clin Rehabil. (2018) 32(7):985–93. 10.1177/0269215518768385 29627995

[17] Carpenter CR Bassett ER Fischer GM Shirshekan J Galvin JE Morris JC. Four sensitive screening tools to detect cognitive dysfunction in geriatric emergency department patients: brief Alzheimer’s screen, short blessed test, Ottawa 3DY, and the caregiver-completed AD8. Acad Emerg Med. (2011) 18(4):374–84. 10.1111/j.1553-2712.2011.01040.x 21496140 PMC3080244

[18] Braun V Clarke V. Using thematic analysis in psychology. Qual Res Psychol. (2006) 3(2):77–101. 10.1191/1478088706qp063oa

[19] Nutbeam D McGill B Premkumar P. Improving health literacy in community populations: a review of progress. Health Promot Int. (2017) 33(5):901–11. 10.1093/heapro/dax015 28369557

[20] Fleisher L Buzaglo J Collins M Millard J Miller SM Egleston BL Using health communication best practices to develop a web-based provider–patient communication aid: the CONNECT™ study. Patient Educ Couns. (2008) 71(3):378–87. 10.1016/j.pec.2008.02.017 18417312 PMC2509582

[21] Fleming J Brayne C. Cambridge city over-75s cohort study c. Inability to get up after falling, subsequent time on floor, and summoning help: prospective cohort study in people over 90. Br Med J. (2008) 337:a2227. 10.1136/bmj.a2227 19015185 PMC2590903

